# Stable polyethylene inlay fixation and low polyethylene wear rate in fixed-bearing total knee arthroplasty at five to six years’ follow-up

**DOI:** 10.1302/2046-3758.135.BJR-2023-0126.R1

**Published:** 2024-05-09

**Authors:** Jonathan H. Jürgens-Lahnstein, Emil T. Petersen, Søren Rytter, Frank Madsen, Kjeld Søballe, Maiken Stilling

**Affiliations:** 1 Department of Clinical Medicine, Aarhus University, Aarhus, Denmark; 2 AutoRSA Research Group, Orthopaedic Research Unit, Aarhus University Hospital, Aarhus, Denmark; 3 Department of Orthopedics, Aarhus University Hospital, Aarhus, Denmark; 4 University Clinic for Hand, Hip and Knee Surgery, Holstebro Regional Hospital, Aarhus, Denmark

**Keywords:** Knee, Arthroplasty, Cemented, Radiostereometry, Polyethylene, polyethylene (PE), Polyethylene (PE) wear, tibial component, knees, Radiostereometric analysis (RSA), total knee arthroplasty (TKA), component migration, femoral components, tantalum, Varus knee

## Abstract

**Aims:**

Micromotion of the polyethylene (PE) inlay may contribute to backside PE wear in addition to articulate wear of total knee arthroplasty (TKA). Using radiostereometric analysis (RSA) with tantalum beads in the PE inlay, we evaluated PE micromotion and its relationship to PE wear.

**Methods:**

A total of 23 patients with a mean age of 83 years (77 to 91), were available from a RSA study on cemented TKA with Maxim tibial components (Zimmer Biomet). PE inlay migration, PE wear, tibial component migration, and the anatomical knee axis were evaluated on weightbearing stereoradiographs. PE inlay wear was measured as the deepest penetration of the femoral component into the PE inlay.

**Results:**

At mean six years’ follow-up, the PE wear rate was 0.08 mm/year (95% confidence interval 0.06 to 0.09 mm/year). PE inlay external rotation was below the precision limit and did not influence PE wear. Varus knee alignment did not influence PE wear (p = 0.874), but increased tibial component total translation (p = 0.041).

**Conclusion:**

The PE inlay was well fixed and there was no relationship between PE stability and PE wear. The PE wear rate was low and similar in the medial and lateral compartments. Varus knee alignment did not influence PE wear.

Cite this article: *Bone Joint Res* 2024;13(5):226–236.

## Article focus

- Polyethylene (PE) wear and PE micromotion.
- PE locking mechanism stability.

## Key messages

- Wear can be measured without a standing reference image.
- There was no relationship between PE wear and PE micromotion.
- The PE liner was well fixed.

## Strengths and limitations

- We evaluated a new method to calculate PE wear.
- We examined the PE inlay only with static radiostereometric analysis and not under dynamic motion.
- Our major limitation is that our study was powered for a different purpose and not wear calculation.

## Introduction

Polyethylene (PE) wear debris is a concern because of its relation to a local inflammation response and osteolysis around the arthroplasty components,^1^ which increase the risk of total knee arthroplasty (TKA) failure and revision surgery.^2^ Multiple retrieval analysis studies of modular fixed-bearing PE inlays with different locking mechanisms in TKA have provided evidence of PE wear debris on both the articular and backside surface.^2-5^ The suggested mechanism of backside wear is micromotion between the PE inlay and the tibial baseplate,^1,3^ which may be the result of an insufficient locking mechanism for the PE inlay. To our knowledge, PE inlay micromotion of TKA has not previously been assessed in vivo.

Radiostereometric analysis (RSA) is an established method to measure migration of implants and predict possible failure.^6^ RSA may also be used to measure migration of the PE inlay.^7,8^ However, the PE inlay is radiolucent and needs to be marked with tantalum beads during surgery for possible radiological visualization and follow-up of PE inlay migration. Inevitably, the PE marker-model becomes a geometrical flat marker-model, which requires a radiological method with high precision, such as RSA. Furthermore, the tantalum markers used in the PE inlay are easily occluded by the femoral or tibial component, which makes marker-based RSA measurements of the PE challenging.^9^

Only a few studies of PE inlay wear of TKA are available.^8,10^ PE wear measurements of TKA can be done reliably using RSA computer models of the femoral and tibial components, but may be affected by a change of the mechanical axis during weightbearing as a result of knee laxity.^11^

The aim of this study was to measure the PE wear (combined backside and articular) at mean six years’ follow-up of an ArCom UHMWPE inlay (Zimmer Biomet) in cemented TKA, measure micromotion of the PE inlay, and evaluate the influence of the anatomical axis on PE wear and tibial component migration.

## Methods

### Ethics

The study was performed in accordance with the Helsinki Declaration.^12^

### Patient cohort description

**Fig. 1:**
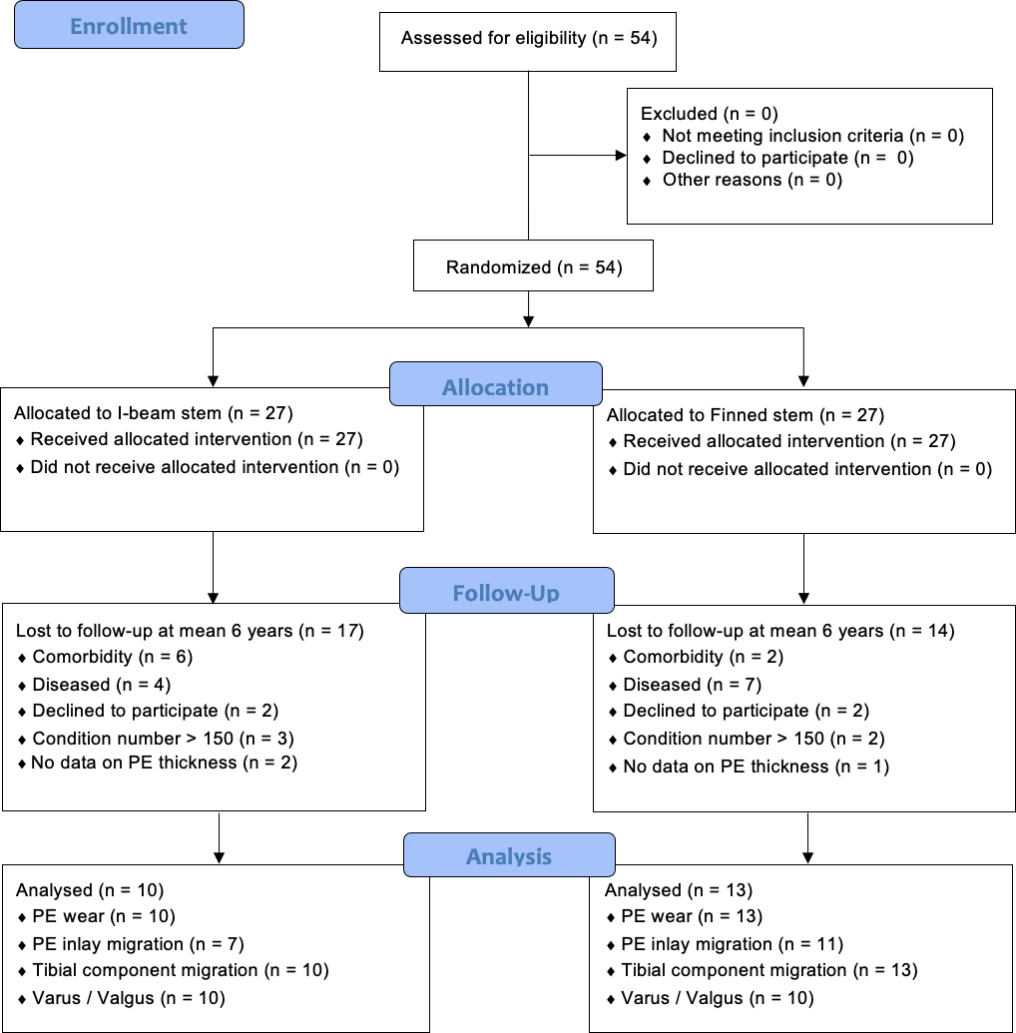
CONSORT flowchart to mean six years’ follow-up. PE, polyethylene.

This study was nested in a study carried out between January 2005 and December 2007, which included 54 patients with primary osteoarthritis (OA) of the knee from a single-centre patient-blinded randomized controlled clinical trial. See Stilling et al^13^ for exclusion and inclusion details. PE inlay migration combined with PE wear has not been analyzed in this group. Randomization in blocks of six patients (three per group) was done by drawing labels from a box, and the labels were then concealed in 54 consecutively numbered closed envelopes. All eligible patients received allocation intervention (Figure 1, Table I). The inclusion criteria were primary OA of the knee, age above 70 years, informed consent, and only one knee operated. The exclusion criteria were severe neuromuscular or vascular disease of the lower limbs, known osteoporosis, previous proximal tibial osteotomy, or another major knee surgery. Patients were followed until a cross-sectional mean of six years’ (five to seven) follow-up, and results for bone mineral density changes and detailed migration pattern of the tibial component have been published previously.^13^

### Patient demographic data

Patient follow-up and analyses are outlined in the CONSORT flowchart (Figure 1). The preoperative baseline characteristics were similar between stem groups and therefore reported for the total group (Table I). All patients had stable knees upon clinical testing of the medial-lateral (valgus/varus) knee stability (0° to 4°).

**Table I.** Patient baseline characteristics and six-year parameters for varus/valgus knee alignment during stance (determined by the absolute deviation from 175°) and lateral and medial compartment polyethylene wear (measured as penetration (minimum joint space width)) in mm/years (stem groups combined).

Characteristic	Mean	95% CI	Range
Age, yrs	82.9	80.9 to 84.9	77 to 91
BMI, kg/m^2^	28.5	26.5 to 30.5	20 to 37
Sex, n	13 M / 10 F		
Varus knee (n = 8), °	2.02	0.63 to 3.41	0.43 to 4.59
Valgus knee (n = 15), °	1.48	0.87 to 2.10	0.10 to 3.63
PE wear lateral (n = 23), mm/year	0.08	0.07 to 0.10	0 to 0.16
PE wear medial (n = 23), mm/year	0.07	0.05 to 0.09	0 to 0.18

PE data were missing for the finned stem (n = 3) and I-beam stem (n = 5) groups.

CI, confidence interval; PE, polyethylene.

### Implants

**Fig. 2:**
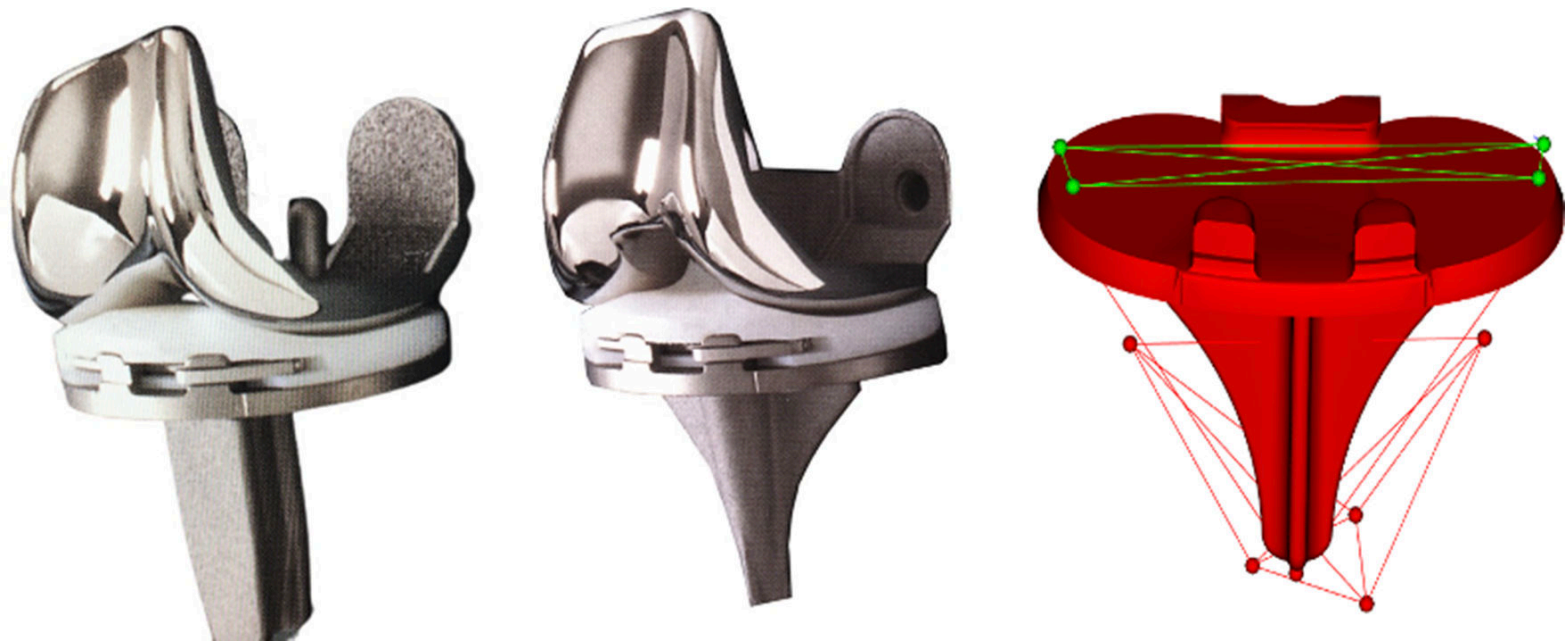
The I-beam block-stem tibial component (Maxim Tibial Tray Interlock, Zimmer Biomet, USA) to the left and the finned stem in the centre. Example of polyethylene marker (green) distribution to the right. The locking mechanism (anterior horizontal split-lock) is the same for both the I-beam and finned stem tibial components.

The cobalt-chromium modular Maxim Tibial Tray Interlock cruciate-retaining components (Zimmer Biomet) had either an I-beam block stem or a Finned stem (Figure 2). Both stem types were 4 cm long and fixed to the tibial baseplate (non-modular). Both tibial components were fixed in the bone by vacuum-mixed Palacos R bone cement (Heraeus Medical, Germany) applied under the baseplate while the stem was fixed press-fit (without cement) in the proximal tibia. The femoral component (Maxim cobalt-chromium) and the patella resurfacing PE component were fixed by Palacos R bone cement. The tibial PE insert was a modular component of gamma sterilized ArCom (Zimmer Biomet) ultra-high molecular weight PE fixed with the similar anterior locking splits in both the I-beam block stem and Finned stem components.

### Surgery

All patients were operated on by four experienced knee surgeons in a theatre with laminar air flow. A tourniquet was applied, and an anterior midline incision was used. The posterior cruciate ligament was retained in all cases. In both groups, the proximal tibia was cut using the same extramedullary guide aiming for a perpendicular cut in the frontal plane and a posterior slope of 3°. The cut surfaces were cleaned by high-pressure lavage before cementing. Five to six tantalum beads (1 mm) (Wennebergs Finmek, Sweden) were inserted in the proximal tibia intraoperatively, and four to six markers were inserted in the PE using a template (Figure 2). All patients received a draining tube in the joint for approximately 24 hours. Preoperatively, all patients were prophylactically given antibiotics (2 g dicloxacillin intravenously). Thromboprophylactics were given postoperatively with one daily dose of 2.5 mg fondaparinux subcutaneously for five to seven days. The patients were mobilized on the first postoperative day and allowed weightbearing as tolerated, with the assistance of two crutches for the first six weeks. The in-hospital stay varied between four and six days.

### Radiostereometric analysis

**Fig. 3:**
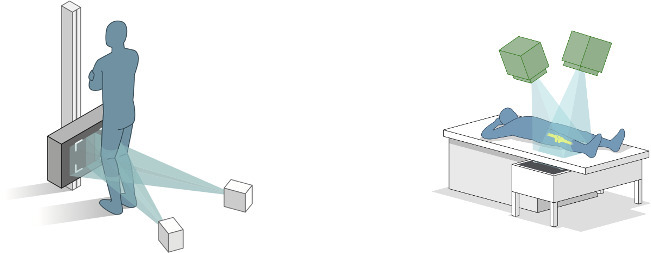
The radiostereometric analysis setup for the standing examination (left) and supine examination (right).

**Fig. 4:**
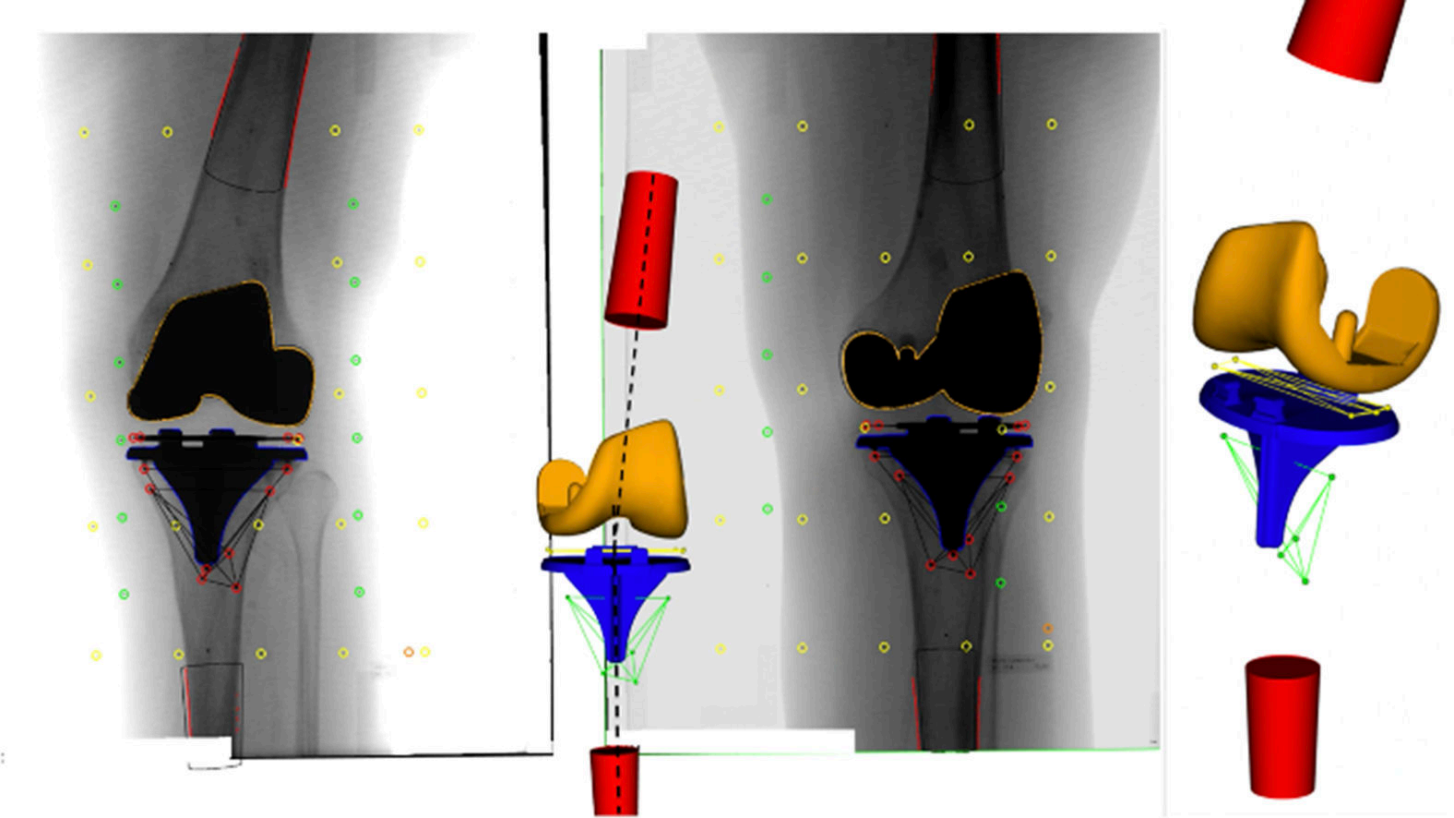
Stereoradiograph with projections of the surfaces of the femoral (orange) and tibial (blue) components, the tantalum markers in the polyethylene inlay (yellow marker model), and in the tibial bone (green marker model). The red cones represent the femur and tibia bones and their coordinate systems (y-axis) for estimation of the anatomical knee axis.

For RSA, we used a fully digitized standard RSA setup (FCR Profet CS; Fujifilm, Denmark) with two synchronized ceiling fixed roentgen tubes (Arco-Ceil/Medira; Santax Medico, Denmark) angled 40° on each other and an unfocused uniplanar carbon calibration box (Box 24; Medis Specials, Netherlands). The first stereoradiographs were obtained two to three days postoperatively with the patients placed supine with the operated knee aligned parallel to the calibration box (y-axis) in a foam positioner at 30° knee flexion (reference examination, non-weightbearing) (Figure 3). Cross-sectionally at mean six years’ follow-up, standing (weightbearing) stereoradiographs with 30° knee flexion and the leg in parallel alignment to the calibration box (y-axis) were obtained in addition to supine stereoradiographs (Figure 3). Double weightbearing RSA examinations were performed for 17 patients for assessment of precision. Analysis was performed with Model-Based RSA version 4.0 (RSAcore, Netherlands) using computer-aided design (CAD) implant surface models (10.000 polygons) and tantalum beads placed in the PE (Figure 4).

### Polyethylene wear

**Fig. 5:**
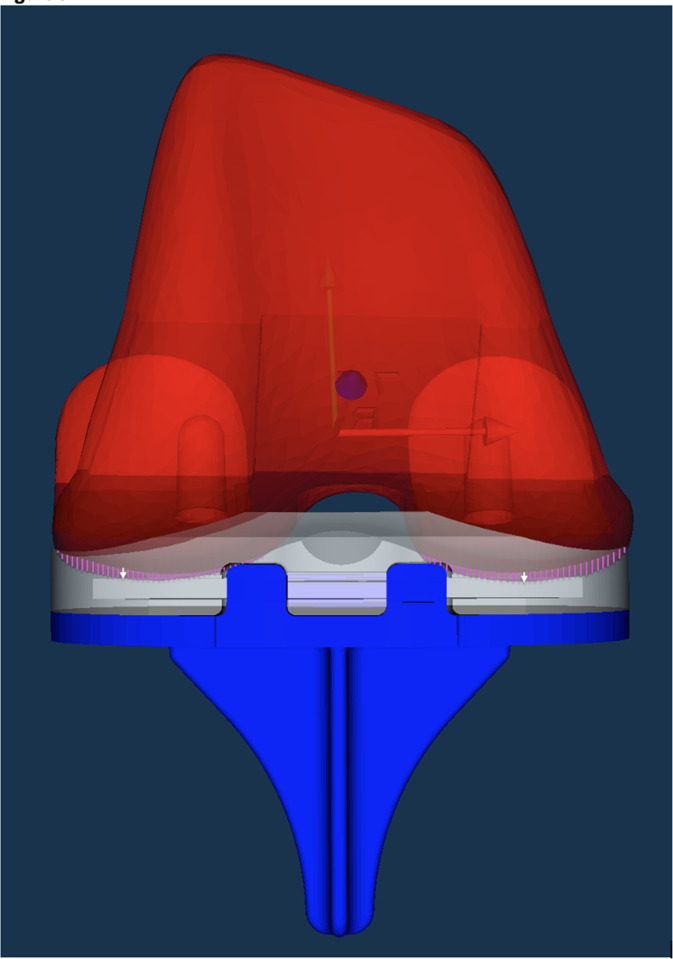
View of the computer-aided design models of the femoral component (red), the polyethylene (PE) inlay (white), and the tibial component (blue). The white arrows indicate the point of deepest penetration of the femoral component into the PE inlay at mean six years’ follow-up (minimum joint space width), which reflects the mean PE wear (mm) in the medial and lateral compartments separately (n = 23). Pink shades indicate penetration area.

CAD models for the femur, tibia, and PE inlay were provided by the manufacturer (Zimmer Biomet). The manufacturer states a manufacturing tolerance of ± 0.13 mm for the PE inlay. The positions of the femoral and tibial components were estimated with model-based RSA for the mean six years’ follow-up standing stereoradiographs. The tibial component and PE inlay models were merged into one model and represented in the same coordinate system, which enables positioning of the radiolucent PE model according to the RSA estimated tibial model pose at midterm (Figure 5). We assumed that the PE liner and femoral TKA component aligned perfectly postoperatively, meaning that there was no penetration of the femoral component into the PE inlay and no lift-off. This assumption must be made since we do not have postoperative weightbearing stereoradiographs. The wear at midterm was calculated as the greatest penetration of the femoral model into the PE inlay model, assuming that there was no penetration of femoral model into the PE inlay model postoperatively. The wear value represents a combination of articular and backside wear because the backside of the PE inlay model is always attached to the tibial baseplate in the model. The PE inlay was separated along the z-axis into a medial and lateral side, for which the wear was estimated separately. Wear was reported as mean wear (mm) of the PE inlay and as the wear-rate per year (mm/year) for the lateral and medial compartments, respectively. It is possible to use both non-weightbearing and weightbearing stereoradiographs to measure PE wear, but comparisons of wear should be made between studies that used the same setup. van Ijsseldijk et al^8^ found that differences between weightbearing and non-weightbearing PE inlay penetration were 0.28 mm medially and 0.20 mm laterally. We used the triangulated surface models for the femur, tibia, and PE components to measure the depth of the maximum penetration point of the femoral component into the PE inlay in both the medial and lateral compartments as a measure of PE wear at midterm with reference to expected neutral component alignment and no postoperative PE wear. We assume perfect postoperative alignment between the femoral component and PE inlay. The method is applicable for PE wear of any TKA at midterm based on component positions on weightbearing RSA when models of the femoral, tibial, and PE inlay components, aligned in the same coordinate system, can be provided by the manufacturer.

### Tibial component migration

The fixed rigid body reference for measurement of tibial component migration (Table II) was a tibial bone marker model with a range of five to six markers. The point of tibial component migration measurement was the centre of gravity of the 3D implant model. The coordinate system was oriented according to the calibration box in the RSA setup, since all knees were aligned perfectly with the y-axis of the calibration box. Migration analyses were made with the postoperative non-weightbearing stereoradiographs as reference (baseline). Translations (implant migration along the axes) were expressed as x-translation (medial and lateral migration), y-translation (cranial ‘lift-off’ and caudal ‘subsidence migration’), z-translation (anterior and posterior migration), and total translation $TT=\surd \left({xt}^{2}+{yt}^{2}+{zt}^{2}\right)$ . Rotations (implant rotation about the axes) were expressed as x-rotation (anterior and posterior tilt), y-rotation (internal and external rotation), and z-rotation (medial and lateral tilt), as well as total rotation $TR=\surd \left({xr}^{2}+{yr}^{2}+{zr}^{2}\right)$ . Positive and negative values were defined from the ‘right-handed’ coordinate system, with appropriate corrections made for left knees. Lastly, maximum total point motion, the point in the implant model that moved the most, was used as a combined migratory measure for both rotation and translation.

**Table II.** Polyethylene inlay and tibial component migration from postoperative to mean six years postoperatively for the I-beam and Finned stem groups combined.

Variable	PE inlay migration (n = 18)		Tibial component migration (n = 23)	
	**Mean**	**95% CI**	**Mean**	**95% CI**
x-translation (+ lateral / - medial), mm	-0.01	-0.06 to 0.04	0.03	-0.03 to 0.08
y-translation (+ lift off / - subsidence), mm	-0.01	-0.04 to 0.02	0.03	-0.03 to 0.08
z-translation (+ anterior / - posterior), mm	-0.03	-0.10 to 0.05	-0.23	-0.50 to 0.02
x-rotation (+ anterior / - posterior tilt),°	-0.35	-0.62 to -0.08	-0.40	-0.78 to -0.01
y-rotation (+ internal rotation / - external rotation),°	-0.12	-0.55 to 0.31	-0.04	-0.35 to 0.28
z-rotation (+ varus / - valgus),°	0.02	-0.03 to 0.07	-0.05	-0.15 to 0.06
Total translation	0.17	0.13 to 0.21	0.47	0.26 to 0.68
Total rotation	0.64	0.46 to 0.82	0.91	0.56 to 1.26
Maximum total point motion	0.39	0.30 to 0.49	0.90	0.53 to 1.27

CI, confidence interval; PE, polyethylene.

The upper limit for mean error rigid body fitting (marker stability) was 0.35 mm, as specified by previous recommendations.^14^ The condition number (CN) refers to the dispersion of the bone markers in the bone, and was mean 70 (standard deviation (SD) 35; 27 to 133) for the tibial bone model. The CN magnitude correlated to the number of markers detected. Thus, fewer markers generated a higher CN, and consequently a less reliable reference for determining migration. For RSA of TKA, a CN for the tibial bone marker model above 150 is considered unreliable. If fewer than three markers were detected or a CN of above 150 was present, the stereoradiographs were considered unreliable and excluded.^14^

### Polyethylene inlay migration

The PE inlay was represented by a marker model based on at least three tantalum beads in the PE inlay. In the event that not all tantalum beads were visible in all three RSA images, a mean marker model was created and used.^9^ The fixed reference for measurement of the PE inlay migration was the tibial component CAD model. The coordinate system was oriented according to tibial component. PE inlay migration analyses were made with the postoperative stereoradiograph as reference (baseline). The translation, rotation, and maximum total point motion were calculated in the same way as for the tibial component. If a stereoradiograph did not meet the CN and marker stability conditions as defined earlier in this article (CN < 150, mean error < 0.35 mm), the patient was excluded (n = 8).

### Varus and valgus measurement of the knee

In order to determine the anatomical axis between the femur and tibia, we applied an elementary geometric-shape model (a cone) to the femoral and tibial bones by use of the EGS-module in ModelBased-RSA 4.0 (RSAcore) on the weightbearing mean six years’ follow-up stereoradiographs. A neutral anatomical axis was defined as an angle of 175°, an angle above 175° was defined as varus, and an angle below 175° was defined as valgus.^15^

### RSA precision

Precision was calculated from weightbearing double examination stereoradiographs taken at mean six years’ follow-up for tibial component migration (Table III), PE inlay (Table IV) migration, and wear calculations (Table V) (I-beam = 10, Finned stem = 13). The difference between the two stereoradiographs represents the systematic error (bias), and the SD represents the precision. We calculated both signed and unsigned values to account for the negative and positive numbers cancelled. The clinical precision was expected to fall within the prediction interval (PI) (1.96 * SD). ModelBased-RSA has been shown to have a mean accuracy of -0.009 mm (SD 0.094) for translations.

**Table III.** Repeatability of polyethylene inlay migration (both stem groups) as signed values from radiostereometric analysis double examinations.

Variable	Translation, mm			Rotation, °		
	**x-translation** **(+ lateral / - medial)**	**y-translation** **(+ lift off / - subsidence)**	**z-translation** **(+ anterior / - posterior)**	**x-rotation** **(+ anterior / - posterior tilt)**	**y-rotation** **(+ internal rotation / - external rotation)**	**z-rotation** **(+ varus / - valgus)**
Mean difference	0.01	0.00	0.03	0.09	0.03	0.00
Precision (SD)	0.04	0.06	0.15	0.24	0.24	0.08
PI 95% (1.96 × SD)	0.08	0.11	0.30	0.46	0.45	0.15
Min. difference	-0.07	-0.10	-0.27	-0.25	-0.48	-0.20
Max. difference	0.09	0.08	0.31	0.42	0.53	0.10

PI, prediction interval; SD, standard deviation.

**Table IV.** Repeatability of polyethylene inlay migration (both stem groups) as absolute values from radiostereometric analysis double examinations.

Variable	Translation, mm			Rotation, °		
	**x-translation** **(+ lateral / - medial)**	**y-translation** **(+ lift off / - subsidence)**	**z-translation** **(+ anterior / - posterior)**	**x-rotation** **(+ anterior / - posterior tilt)**	**y-rotation** **(+ internal rotation / - external rotation)**	**z-rotation** **(+ varus / - valgus)**
Mean difference	0.04	0.04	0.11	0.19	0.16	0.06
Precision (SD)	0.02	0.04	0.10	0.16	0.17	0.04
PI 95% (1.96 × SD)	0.04	0.08	0.20	0.32	0.33	0.09
Min. difference	0.00	0.01	0.00	0.01	0.01	0.00
Max. difference	0.09	0.15	0.31	0.57	0.53	0.20

PI, prediction interval; SD, standard deviation.

**Table V.** Repeatability of wear calculations (mJSW), signed and absolute values.

Variable	Signed, mm		Absolute, mm	
	Lateral compartment	Medial compartment	Lateral compartment	Medial compartment
Mean difference	-0.01	0.02	0.12	0.10
Precision (SD)	0.17	0.17	0.12	0.13
PI 95% (1.96 × SD)	0.33	0.33	0.23	0.26
Min. difference	-0.43	-0.33	0.00	0.00
Max. difference	0.35	0.53	0.43	0.53

PI, prediction interval; SD, standard deviation.

### Clinical follow-up

The patients were seen for clinical examination preoperatively, and at a mean of six years postoperatively. Clinical data (American Knee Society Scores (AKKS))^16^ collection was conducted unblinded by four surgeons. The AKSS was used to quantify the knee stability.

### Post-hoc power analysis

The study was nested in a randomized study, in which a pre-study sample size calculation deemed a need for 22 patients per tibial stem group for evaluation of tibial component migration.

### Statistical analysis

Normal distribution of continuous data was assessed using QQ plots. For PE wear and RSA precision, the Mann-Whitney U test was used to determine statistical differences between groups. Spearman’s rank correlation was used for the correlation analysis. Other data were reported using means and 95% confidence intervals (CIs). We report tibial component migration, PE wear, and PE inlay migration for both stem groups combined, since no statistically significant difference could be found. Stata IC version 16 (StataCorp 2019; Stata Statistical Software: Release 16, StataCorp, USA) was used for statistical analysis. The significance level was set at p = 0.05.

## Results

### Polyethylene wear

The combined articulate and backside mean wear rate of 0.08 mm/year (95% CI 0.06 to 0.09 mm/year) was below the clinical precision limit (Table V). The PE wear of the medial and lateral compartment was similar (p = 0.110, Spearman test) (Table I). The manufacturing tolerance for the PE inlay was ± 0.13 mm. There was no correlation between PE wear and PE inlay migration (rho = 0.35, p = 0.153), nor was there any correlation between PE wear and knee alignment (rho = -0.18, p = 0.402, both Spearman test).

### Polyethylene inlay migration

The combined PE inlay maximum total point motion migration in relation to the tibial component as reference was 0.39 mm (95% CI 0.30 to 0.49) MTPM and 0.17 mm (95% CI 0.13 to 0.21) total translation (Table II). The mean PE inlay external rotation in relation to the tibial component was -0.12° (95% CI -0.55° to 0.31°), while no PE inlay moved had an external rotation above the precision limit (PI 95% 0.47°) (Table II and Table IV). Mean PE inlay subsidence was -0.01 mm (95% CI -0.04 to 0.02), and no PE inlay subsided above the precision limit PI (95% 0.05 mm) (Table II and Table IV). The mean x-rotation of the PE inlay was -0.35° (95% CI -0.62° to -0.08°) (Table II).

None of the PE inlays had a migration above the precision limit of 0.14 mm along the y-axis.

There was no significant correlation between PE inlay migration and knee alignment (rho 0.12, p > 0.624, Spearman), and no correlation between PE inlay migration and tibial component migration (rho = -0.20, p > 0.413, Spearman). However, there was a negative correlation between varus alignment and TT of the PE inlay (rho = -0.75, p = 0.052, Spearman).

### Tibial component migration

**Fig. 6:**
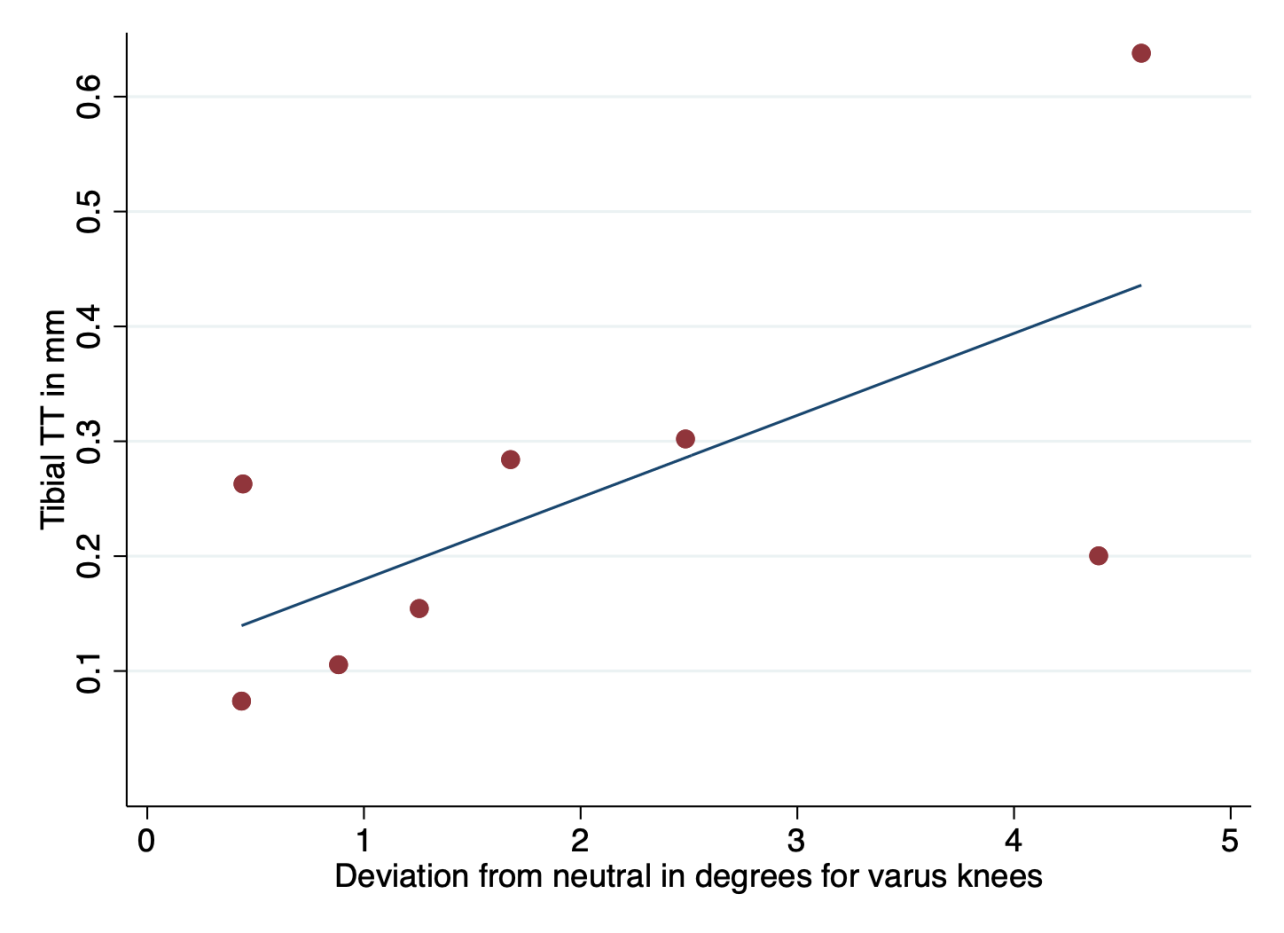
Graph showing the correlation between the varus deviation from the neutral anatomical knee axis (°) and the total translation of the tibial component (rho = 0.74, p = 0.04). TT, total translation.

At six years’ follow-up, the mean combined stem group tibial component maximum total point motion and total translation in relation to the tibial bone markers as reference was 0.90 mm (95% CI 0.53 to 1.27) and 0.47 mm (95% CI 0.26 to 0.68), respectively (Table II). There was a significant correlation between tibial component migration (total translation) and the amount of varus deviation (°) from the neutral anatomical axis of the knee (rho = 0.74, p = 0.04) (Figure 6).

### Varus/valgus alignment

There were no knees in perfect neutral alignment on weightbearing stereoradiographs at mean six years’ follow-up. Mean varus alignment was 2.02° (95% CI 0.62 to 3.41), and mean valgus alignment was 1.48° (95% CI 0.88 to 2.10) (Table I).

### Method precision

For both stem groups combined, the absolute mean difference of PE inlay translations and rotations between double examination stereoradiographs were ≤ 0.11 mm and < 0.19° (Table IV). The precision limit for PE inlay migration was 0.20 mm and 0.33° (Table IV). Absolute combined tibial stem group mean difference of translations and rotations was < 0.08 mm and < 0.29°, respectively. The precision limit (95% PI) for tibial migration was 0.18 mm and 0.62°. Mean absolute combined wear difference for the lateral compartment was 0.12 mm, and 0.10 mm for the medial compartment (Table V).

## Discussion

### Key findings

The key findings at mean six years’ follow-up were similar PE wear in the medial and lateral compartment of mean 0.08 mm/year and no measurable migration of the PE inlay. There was no effect of knee alignment on PE wear or PE inlay migration, but a moderate correlation between the degree of varus alignment and tibial component migration.

### Polyethylene wear

RSA is a valuable tool to detect early implant component migration as a proxy measure for later aseptic arthroplasty component loosening as well as PE wear in total hip arthroplasty (THA).^6^ New EU regulations requiring a phased introduction of new prostheses suggest fast, reliable, and precise techniques to evaluate all aspects of a prosthesis.^17^ Previous studies measured PE inlay wear by comparing worn retrieved PE bearings with new bearings,^18,19^ using micro-CT scans to measure the points of deepest penetration in mm, and visual comparison using the scoring system developed by Hood et al.^20^ A phantom study using stereoradiogaphs and Model-Based RSA showed an accuracy of 0.1 mm and a precision of 0.2 mm, influenced by knee flexion and prosthesis type, of the fixed-bearing Duracon TKA and the fixed-bearing Triathlon TKA, both from Stryker.^11^ Gascoyne et al^10^ measured wear in TKA (Triathlon Cruciate Retaining, Stryker; PE: UHMWPE) with a similar method to the one used in the present study, and they reported wear rates of 0.222 mm/year in the medial compartment and 0.236 mm/year in the lateral compartment with a precision of 0.299 mm. In another study, Teeter et al^21^ reported (TKA: all posterior-stabilized, cobalt-chromium, Genesis II; PE: UHMWPE) medial wear rates of 0.052 mm/year and lateral wear rates of 0.047 mm/year. Stilling et al^13^ have previously reported a wear rate of 0.21 mm/year (TKA: Maxim Cruciate Retaining, Zimmer Biomet; UHMWPE) for the I-beam stem group and of 0.10 mm/year for the Finned stem group using a simple method that measured a mean (centred) y-axis translation difference of the femoral component on the tibial component from postoperative to mean six years’ follow-up. In the present study, we calculated a more precise mJSW, for both the medial and lateral compartments, and found a lower wear rate in both compartments compared to the previous report. Our PE wear measurement method found the point of deepest penetration in three dimensions, rather than only two, and we did not rely on a reference examination. Theoretically, the differences in reported PE wear of TKA in the literature could be explained with various prosthesis designs, PE types, sterilization methods, shelf age, and differences in the weightbearing setup and wear measurement methods. Teeter et al^21^ and Gascoyne et al^10^ used reverse-engineered models of the femoral and tibial components as well as the PE inlay using micro-CT or a laser scanner respectively, thus accounting for manufacturing errors. Gascoyne et al^10^ and the current study used CAD models of the tibial and femoral components and the PE inlay, which were provided by the manufacturer. Gascoyne et al^10^ obtained weightbearing steroradiographs in full knee extension, while Teeter et al^21^ reported mean wear rate based on different knee flexion angles, compared to the current study in which stereoradiographs were taken in 30° knee flexion. The shelf age of the PE inlays used in the present study are not reported. Compared to the current study, Teeter et al^21^ and Gascoyne et al^10^ used different prosthesis designs, different weightbearing setups, and different means of acquiring CAD models of the prosthesis and PE inlay. Teeter et al^21^ used PE that was sterilized with ethylene oxide, while Gascoyne et al^10^ used a non-crosslinked insert; the sterilization method was not reported. In the current study the PE inlays were gamma ray-sterilized, which is known to give less PE wear in clinical studies of THA.^22^ The PE-related differences in particular may explain the differences in reported wear rates. Weightbearing stereoradiograph examinations is recommended for PE wear measurement of TKA.^8^ However, the most optimal knee flexion angle, which would capture the most pronounced PE wear (mJSW) on image recording, is not known. We used a standardized set-up, in which all patients had a mean knee flexion angle of 30° on weightbearing stereoradiograph recordings, which is similar to previous publications.^7^ In a future study, the most optimal knee flexion angle for measurement of linear PE wear could be investigated during changes in a dynamic stereoradiograph setup.^7^

### Polyethylene migration/stability of the polyethylene locking mechanism

Factors influencing backside wear of the PE inlay include the component material (cobalt-chromium or titanium), the surface roughness, and the design and stability of the PE inlay locking mechanism.^23^ A previous study investigated the influence of PE inlay micromotion on PE wear debris based on retrieval studies and wear patterns observed on the backside of the PE inlay ex vivo.^24^ Marks found on the backside of the PE inlay in a polyethylene fixed-bearing TKA design indicated a rotary micromotion pattern, with a rotation around the y-axis.^24^

To our knowledge, the present study is the first to measure PE inlay migration in vivo using RSA. We found little PE inlay subsidence (mean -0.01 mm) and little PE inlay external rotation (mean -0.12°), which was less than the precision limit. We found a rotation along the x-axis of the PE inlay, which indicates backside wear. Since we have not found any subsidence, the rotation around the x-axis does not to seem relevant, or our method to detect subsidence is not sensitive enough. Furthermore, we did not find any correlation between PE inlay migration and PE wear, which indicates a stable PE inlay and a stable locking mechanism. However, we only examined the patients while they were standing, and we cannot conclude on the stability of the locking mechanism and PE inlay during dynamic conditions, i.e. gait, which may be relevant to investigate since we know of at least one case of a failing locking mechanism.^25^ It is difficult to visualize the polyethylene markers in a standardized static RSA set-up, therefore a marker-based dynamic evaluation of PE inlay migration would be even more challenging. Our study provides substantial evidence that the locking mechanism of the Maxim TKA components is safe, and that liner movement does not contribute to PE wear and probably reduces the risk of aseptic loosening. Backside PE wear may be of less importance than articulate PE wear.

### Tibial component migration

The tibial component migration of the two stem types was described extensively in another study using non-weightbearing stereoradiographs.^13^ We found similar tibial component migration magnitude on weightbearing stereoradiographs and, in addition, a correlation between increasing anatomical varus axis and increasing tibial component total translation, which is in line with the current literature.^21^

### Effect of knee axis on polyethylene wear and polyethylene migration

Neutral mechanical femoral-tibial alignment is considered to be the gold standard in TKA, with ‘neutral’ defined as +/-3° from 180°.^26^ Other knee alignment types include anatomical and kinematic alignment.^26^ Yet, there is no consensus regarding which type of alignment is preferable for good long-term TKA survival. However, the literature tends to indicate that increased varus malalignment leads to increased tibial component migration and increased PE wear.^27,28^ We found no correlation between knee alignment and PE wear, which may be due to the small number of subjects or because we used the anatomical axis instead of the mechanical axis.

### Clinical results

We used the AKSS to determine if any of the included patients had medial-lateral knee instability, since instability above 5° could affect the PE wear measurements.^8^ None of our patients had a medial-lateral knee instability above 5°.

One important limitation is that we only had 23 patients available, due to a high dropout rate during follow-up of the original randomized study, primarily because of comorbidity or death in a rather old study group. Furthermore, we were not able to measure the mechanical axis, as no hip-knee-ankle images were available. Instead, we measured the anatomical axis using cones on the femoral and tibial bones by use of EGS RSA. This could influence our correlation results between PE inlay migration and knee alignment and tibia component migration. In addition, static positional RSA recordings may not represent the true migration of PE inlays. We did not have any pronounced varus or valgus legs, which may weaken the conclusion. We assumed perfect PE inlay and femoral condyle alignment postoperatively, which may not always be the case.

A strength of the current study was our choice of wear measurement method. This method does not rely on a reference examination – as only one follow-up stereoradiograph is needed – and it examines articulate PE wear as well as backside PE wear.

In conclusion, PE wear was not influenced by PE inlay migration, tibial component migration, or knee alignment measured on static weightbearing stereoradiographs at mid-term follow-up. Increased varus knee alignment led to increased tibial component total translations.

## Data Availability

The datasets generated and analyzed in the current study are not publicly available due to data protection regulations. Access to data is limited to the researchers who have obtained permission for data processing. Further inquiries can be made to the corresponding author.
